# MetExploreViz: web component for interactive metabolic network visualization

**DOI:** 10.1093/bioinformatics/btx588

**Published:** 2017-09-15

**Authors:** Maxime Chazalviel, Clément Frainay, Nathalie Poupin, Florence Vinson, Benjamin Merlet, Yoann Gloaguen, Ludovic Cottret, Fabien Jourdan

**Affiliations:** 1 Toxalim, Université de Toulouse, INRA, Université de Toulouse 3 Paul Sabatier, Toulouse, France; 2 MedDay Pharmaceuticals, Paris, France; 3 Glasgow Polyomics, College of Medical, Veterinary and Life Sciences, University of Glasgow, Wolfson Wohl Cancer Research Centre, Gascube Campus, Bearsden, UK; 4 LIPM, Université de Toulouse, INRA, CNRS, Castanet-Tolosan, France

## Abstract

**Summary:**

MetExploreViz is an open source web component that can be easily embedded in any web site. It provides features dedicated to the visualization of metabolic networks and pathways and thus offers a flexible solution to analyse omics data in a biochemical context.

**Availability and implementation:**

Documentation and link to GIT code repository (GPL 3.0 license) are available at this URL: http://metexplore.toulouse.inra.fr/metexploreViz/doc/

## 1 Introduction

Visualization is a much-needed approach in the era of untargeted omics since it enables discovering new insights in large molecular data corpus (Callaway, 2016). For metabolic studies, challenge resides in creating visual representations of all, or part of, the thousands of reactions and metabolites constituting genome scale metabolic networks.

Visualizing metabolic networks necessitates specific graphical features that are not necessarily available in generic network visualization tools (Bastian *et al.*, 2009; Cline *et al.*, 2007) or web based tools (Franz *et al.*, 2016). Resources are available to perform omics data mapping on metabolic pathways (Caspi *et al.*, 2016; Kanehisa *et al.*, 2014) or on overview of metabolism (Noronha *et al.*, 2017), but they mostly rely on manually drawn maps which cannot integrate network updates or be used for new metabolic reconstructions (Karp *et al.*, 2016). Automatic drawing is thus needed to fit current growing research activity in the field.

Current trend in web development is oriented toward modular architectures allowing assembling specialized web components. In this context, we designed MetExploreViz as an easy to install open source web component that can quickly and easily be embedded in any website. Only few lines of code are required to add MetExploreViz to any web page (see installation section at http://metexplore.toulouse.inra.fr/metexploreViz/doc/).

## 2 Metabolic network visualization features

In order to make MetExploreViz a versatile solution, it is possible to import networks in JSON format. These networks can for instance be retrieved by using MetExplore web services (Cottret *et al.*, 2010). Drawing is achieved using an animated version of force directed layout implemented in D3.js library upon which MetExploreViz is built. It is also possible to manually edit the representation and then save the drawing in JSON format for further reuse.

**Fig. 1:**
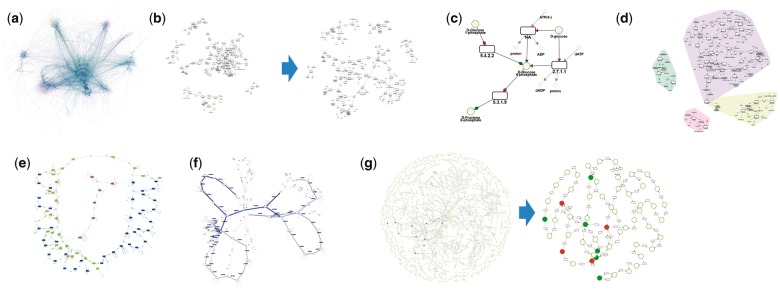
Main MetExploreViz features. (**a**) Visualization of Recon2 human metabolic network containing 7440 reactions (Thiele *et al.*, 2013). (**b**) Sub-network corresponding to glycolysis pathway in cytosol. Highly connected nodes in grey (ATP, ADP, NAD and NADH) on the left network are duplicated to create the sparser representation on the right. (**c**) Zoom in view on the metabolic pathway, reversible reactions are depicted using double-headed arrows. Smaller nodes are duplicated side compounds. (**d**) Compartments where glycolysis is taking place are highlighted using colored convex hulls (e.g. cytosol in top right corner). (**e**) Sub-network with discrete values mapped on reactions. (**f**) Flux values mapped on edges around reactions. (**g**) KEGG network of *Saccharomyces cerevisiae* with metabolomics dataset from (Madalinski *et al.*, 2008) mapped (dark nodes). On the right the sub-network automatically obtained using the algorithm computing the union of lightest paths implemented in MetExploreViz (Color version of this figure is available at *Bioinformatics* online.)

An issue when drawing metabolic networks is the presence of highly connected small molecules like water or CO_2_ which are not necessarily of biochemical interest for data interpretation. To facilitate the visual inspection, these side compounds can be duplicated or deleted in MetExploreViz (Fig. 1b and c).

Each element of a metabolic network can be involved in one or more metabolic pathways and be present in one or several cellular compartments. MetExploreViz offers an original feature to visualize this information using convex hulls (Fig. 1d). Since the force directed algorithm may lead to overlapping hulls, we implemented an algorithm to take these sub-structures into account during the drawing.

MetExploreViz purpose is the visualization of omics data on reactions and metabolites. It is thus possible to import data and map them in a discrete or continuous way using predefined or selected colors (see Fig. 1e and f).

In order to focus on a core sub-network (see Fig. 1g), MetExploreViz implements a sub-network extraction method computing the union of lightest paths between each pairs of mapped (or selected) nodes in the network (Frainay and Jourdan, 2017). Lightest paths aim at avoiding side compounds by weighting nodes according to their degree defined as the number of in and out connections. Classical image exports are available and completed with an SVG export well suited for online representations.

Since MetExploreViz focuses on metabolic networks, it does not provide all the features available in generic network analysis software suites like Cytoscape or Gephi. To allow compatibility with these tools, it is possible in MetExploreViz to export networks in gml and dot formats.

MetExploreViz is provided as an open source web component that only requires few lines of code to be embedded in a web site.

## 3 Discussion and application

MetExploreViz and metabolic pathways oriented representations like KEGG are complementary visualization solutions. MetExploreViz is focused on integrative and flexible network import and representation, while pathway oriented tools are guiding data interpretation toward *a priori* defined metabolic functions.

MetExploreViz is embedded in MetExplore web server (Cottret *et al.*, 2010). But its component oriented architecture allows it to be used in various web servers. For instance, it is available in MetaboLights data repository for metabolomics data sets (Kale *et al.*, 2016). We are also currently working on the integration of MetExploreViz in galaxy pipelines (Giacomoni et al., 2015). MetExploreViz provides complementary features to Escher (King *et al.*, 2015) like sub-network extraction and is not focused on network curation, making it a lightweight component.

The tool has been used for several projects as shown on Figure 1g where metabolomics data from (Madalinski *et al.*, 2008) are represented. The extracted sub-network shown on the figure raised similar conclusions as the ones described in Milreu *et al.* (2014). Tutorial available at http://metexplore.toulouse.inra.fr/metexploreViz/doc/ allows repeating this analysis.
